# Using the SITAR Method to Estimate Age at Peak Height Velocity of Children in Rural South Africa: Ellisras Longitudinal Study

**DOI:** 10.3390/children7030017

**Published:** 2020-03-03

**Authors:** Chris Nembidzane, Maseka Lesaoana, Kotsedi Daniel Monyeki, Alexander Boateng, Phuti J. Makgae

**Affiliations:** 1 Department of Statistics and Operations Research, University of Limpopo, Private Bag X1106, Sovenga, 0727, Polokwane 0699, South Africa; lesaoana.maseka@ul.ac.za; 2 Department of Physiology and Environmental Health, University of Limpopo, Private Bag X1106, Sovenga, 0727, Polokwane 0699, South Africa; kotsedi.monyeki@ul.ac.za (K.D.M.); phutimakgae@gmail.com (P.J.M.); 3 Department of Statistics, University of Cape Coast, PMB Cape Coast-Ghana, Cape Coast CC453, Ghana; alexander.boateng@ucc.edu.gh

**Keywords:** SITAR, age ate peak height velocity, growth spurt

## Abstract

Various studies have been conducted on children using the Ellisras Longitudinal Study Survey in South Africa, but none of these has addressed growth variations of children in this rural location. The aim of this paper is to assess the age at peak height velocity using the superimposition by translation and rotation (SITAR) method for both boys and girls in rural South Africa. The study is part of the on-going Ellisras Longitudinal Study, and has employed secondary data during the period from November 1996 to November 2003. The data was collected biannually in May and November each year. The Ellisras study initially followed a cluster sampling method. All participating children underwent a series of anthropometric measurements of height and weight according to the standard procedures recommended by the International Society for the Advancement of Kinanthropometry. The analysis was done using the SITAR model. Age at peak height velocity for Ellisras rural children was at 14.45 years for boys at 11.82 years for girls. Ellisras rural girls had their age at peak height velocity way earlier than Ellisras rural boys did by an estimated 2.63 years. Ellisras rural children and their growth variations were comparable to other studies.

## 1. Introduction

Human growth studies have been of interest to health professionals and scientists, and as a result, growth was first linked to socio-economic status in the early 19th century [1]. Longitudinal data analysis has long played a significant role in empirical research within the developmental sciences [2]. The human growth curve shows two outstanding periods of accelerated growth—the circumnatal and the adolescent [3]. The circumnatal growth cycle has great velocity, which reaches a maximum at the time of birth [3]. Growth curves are mainly used to analyze and understand the growth of individuals, and the growth of each individual is unique. By its nature, the growth curve consists of a series of highly correlated measurements, and it is important to reduce the dimensionality of the data to simplify comparisons between individual children [4].

Height in puberty involves a growth spurt, the timing and intensity of which varies between individuals [4]. Puberty is a time of substantial biological and psychological changes. [5] One of the hallmarks of puberty is a rapid growth spurt; however, its timing varies between individuals. The impact of pubertal timing on later health outcomes has been of interest in life course epidemiology; however, its measurement can be challenging [5]. Age at peak height velocity (aPHV) offers an objective measure of pubertal timing without having to rely on physical examination or self-reporting. It was found that the mean aPHV for males was 13.6 years and for females was 11.7 years [5].

Superimposition by translation and rotation (SITAR) was applied to evaluate how infant weight and length growth trajectories associate with body composition at three years of age and seven years of age because previous studies have noted that rapid infant weight gain increases the risk for high BMI in children [6]. They found that infant growth trajectories differed by sex, race, and breastfeeding status. Higher overall weight size and weight velocity from birth to two years of age were associated positively with all age three body composition and anthropometry outcomes [6]. The authors of this study concluded that greater average weight size and greater weight velocity in infancy are markers for greater overall body size at three and seven years of age. However, longer average lengths and later weight gain tempo between birth and two years of age may help to establish a leaner body composition by three and seven years of age [6].

SITAR has been used as a growth curve model to quantify the secular trend patterns in height and weight growth in two countries (Japan and South Korea) over 50 years by [7]. SITAR fitted their data well and growth patterns in the two countries changed dramatically over the study period, affecting not only height and weight but also developmental age, in that the growth period advanced in timing and shrank in duration [7]. They further concluded that the secular height trend in these countries represents increased growth in the long bones during infancy, so it can be viewed as the inverse of stunting. There are striking country differences in growth patterns, but they are not easily explained by differences in national income, diet or lifestyle [7].

SITAR was also applied to examine associations of timing of puberty and peak velocity of pubertal height growth with lung function in adolescence and early adulthood [8]. They found that later pubertal age and greater peak velocity were associated with higher FEV_1_ (Forced Expiratory Volume) and FVC (Forced Vital Capacity) at 24 years in both sexes. A one-year increase in pubertal age was associated with a 263-mL higher FVC (95% confidence interval (CI), 167–360 mL) for males (n = 567) and a 100-mL (95% CI, 50–150 mL) higher FVC for females (n = 990). A 1-cm/year increase in peak velocity was associated with 145-mL (95% CI, 56–234 mL) and 50-mL (95% CI, 2–99 mL) increases in FVC for males and females, respectively [8].

Growth variations have not previously been studied for children in rural Ellisras in South Africa, and this is now being addressed in this paper. The analysis of height and weight measurements of children in rural Ellisras have been taken to assess the age at peak height velocity using the SITAR method for both boys and girls.

## 2. Materials and Methods

### 2.1. Study Design

This paper is part of the ongoing Ellisras Longitudinal Study (ELS), which received ethical clearance on 27 July 2001 and subsequently in 2013 by the MREC/P/204/2013:IR. The study will follow secondary analytical longitudinal study using data collected from November 1996 to November 2003. Following the first data collection in November 1996, the data were collected biannually in May and November each year.

### 2.2. Sample and Study Population

#### 2.2.1. Geographical Area

Ellisras is a deep rural area situated within the north-western area of the Limpopo Province, South Africa. The population is about 50,000 people residing in 42 settlements [9]. These villages are approximately 70 km from Ellisras town (23°40 S 27°44 W), now known as Lephalale, which is adjacent to the Botswana border. The Iscor coal mine and Matimba electricity power station are the major sources of employment for many of the Ellisras residents. The remaining workforce is involved in subsistence farming and cattle rearing, while the minority are in the education and civil service sectors. Unemployment, poverty and low life expectancy seem to play a significant role among the communities in rural South Africa, of which the Ellisras rural area is no exception [10,11].

#### 2.2.2. Sample

The ELS initially followed a cluster sampling method [12]. In brief, the study was undertaken at 22 schools (10 preschools and 12 primary schools) randomly selected from 68 schools within the Ellisras area. Birth records were obtained from the school admission register through the assistance of principals in each school. Only those records that were verified against health clinic records were used to determine the ages of potential participants. The South African School Act. 1996 (No. 84 of 1996) stipulates the statistical age norm per Grade X, as the Grade X + 6, implying that the expected age of a child at Grade 1 is 7 years (Grade 1 + 6), with children aged 6 years. The Grade before Grade 1, which is not compulsory, is known as Grade R or Grade 0. Children schooling before Grade 1 are said to be in preschool. In the study by [11], children aged 3–10 years were considered, and each of the 22 selected schools sampled was assigned a grade with the expectation that most of the children in the age category studied would be found in that grade or in a preschool.

For the purpose of this analysis, data collected in November 1996, May 1997, November 1997, May 1998, November 1998, May 1999, November 1999, May 2000, November 2000, May 2001, May 2002, May 2003 and November 2003 were included. A total of 2225 (550 children in preschools with a mean age of 4.4 years and standard deviation of 0.99; and 1675 primary school children with a mean age 8.0 years and standard deviation of 1.11) at baseline were followed throughout the periodic surveys. On average, 1.05% of the participants were permanently lost due to death and 11.47% subjects were lost due to teenage pregnancy, illness and/or migration to urban areas. School dropout was a temporary issue as the affected participants re-joined their studies thereafter.

#### 2.2.3. Anthropometric Measurements

All children underwent a series of anthropometric measurements of height and weight according to the standard procedures recommended by the International Society for the Advancement of Kinanthropometry (ISAK) [13]. Weight was measured on an electronic scale to the nearest 0.1 kg, and a Martin anthropometer was used to measure height to the nearest 0.1 cm.

#### 2.2.4. Quality Control

The survey was carried out over a three-week period by 16 anthropometrists each year, who were required to undertake reliability testing as part of their training. This training was conducted by a level three criterion of ISAK following the guidelines of the ISAK [13]. The absolute and relative values for intra-tester and inter-tester technical error of measurements (% TEM) ranged from 0.12 kg (0.15%) to 0.31 kg (0.36%) for weight and from 0.22 cm (0.12%) to 0.43 cm (0.32%) for height.

### 2.3. Statistical Analysis

All analyses were performed using R version 3.5.3 (R is a free open source programming language, created by Ross Ihaka and Robert Gentleman at the University of Auckland, Newzealand, and is currently developed by the R Development Core Team). The SITAR model was fitted for both boys and girls (combined), and boys and girls separately. SITAR is a mixed effects shape-invariant growth curve model consisting of a mean growth curve along with three transformations (size, tempo and velocity) used to describe how each individual differs from the mean curve. The three SITAR parameters are size, reflecting up/down shift from the mean curve; tempo, reflecting left/right shift (on the age scale), which corresponds to the relative timing of puberty based on aPHV; and velocity, reflecting stretching/shrinking of the age scale and hence describing differences in the rate at which individuals pass through puberty [5]. Cole has detailed the SITAR method in full in [4].

## 3. Results

**Figure 1:**
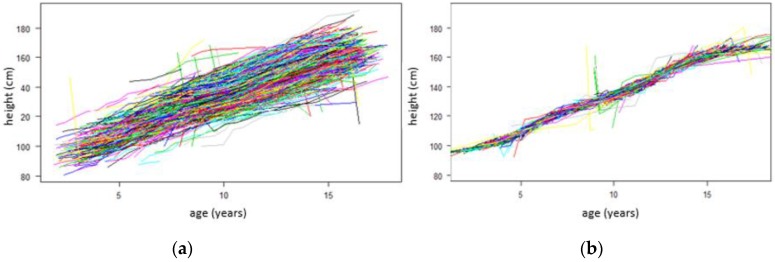
Growth curves for boys and girls. Height growth curves for both boys and girls before SITAR adjustment (**a**) and after SITAR adjustment (**b**). SITAR: Superimposition by Translation and Rotation.

Figure 1 below shows two growth curves for both boys and girls. Figure 1a illustrates growth curves before SITAR adjustment. Figure 1b illustrates growth curves after SITAR adjustment. The adjusted curve involves shifting the curves up/down and rotating the curves (making them steeper or shallower), thus making them superimposed and removing the inter-individual variability.

By looking at the individual curves, it can be observed that some boys and girls are in every case or on every occasion taller and others shorter. In comparison, some start shorter and become taller, whereas others do the opposite.

**Figure 2:**
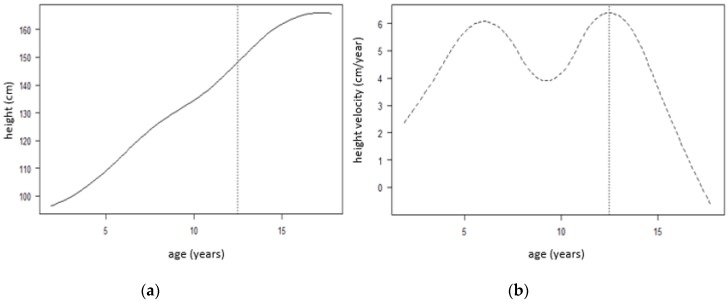
Superimposition by translation and rotation (SITAR) model mean growth curves for both boys and girls. Mean curves, fitted by SITAR model, are illustrated for height growth (**a**) and height velocity (**b**) indicating mean at peak height velocity of 12.51 years (**a**) and mean peak height velocity of 6.39 cm/year (**b**). SITAR: Superimposition by Translation and Rotation.

All boys and girls have a pubertal growth spurt, a time when they grow considerably faster than before or after. Looking at Figure 2b, the timing of the spurt varies between the ages 10 and 15 years.

Figure 2 below shows the SITAR mean growth curve for both boys and girls, which is estimated by taking the adjusted curves (Figure 1b) and fitting a natural cubic spline through the curves. Figure 2a shows that the mean age at peak height velocity is 12.51 years, and Figure 2b shows that the mean peak height velocity is 6.39 cm/year for both boys and girls, marked by a dotted vertical line in both plots.

**Figure 3:**
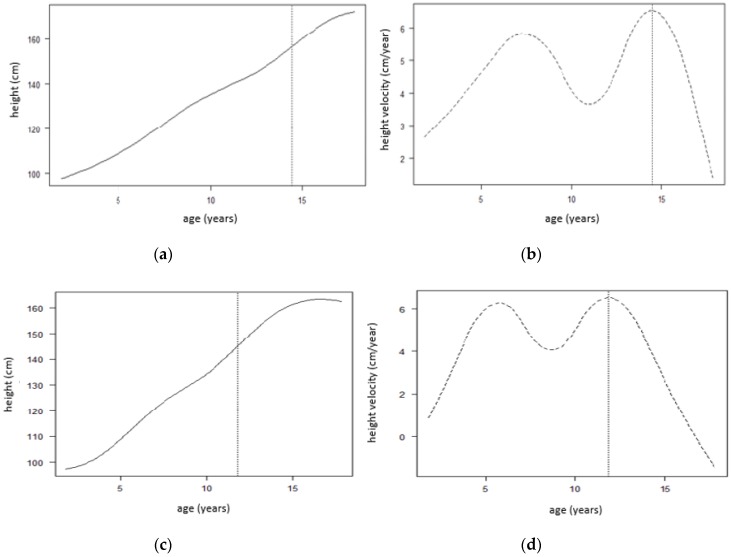
SITAR mean growth for boys and girls (separately). Mean curves, fitted by SITAR model, are illustrated for height growth (**a**,**c**) and height velocity (**b**,**d**) indicating mean at peak height velocity of 14.45 and 11.82 years (**a**,**c**) respectively, and mean peak height velocity of 6.54 and 6.52 cm/year (**b**,**c**) respectively. SITAR: Superimposition by Translation and Rotation.

Figure 3 below shows the SITAR mean growth curve for boys (Figure 3a,b) and girls (Figure 3c,d). Figure 3a shows that the mean age at peak height velocity is 14.45 years, and Figure 3b shows that the mean peak height velocity is 6.54 cm/year for boys only, marked by a dotted vertical line in both plots.

Figure 3c shows that the mean age at peak height velocity is 11.82 years and the Figure 3d shows that the mean peak height velocity is 6.52 cm/year for girls only, marked by a dotted vertical line in both plots.

**Figure 4:**
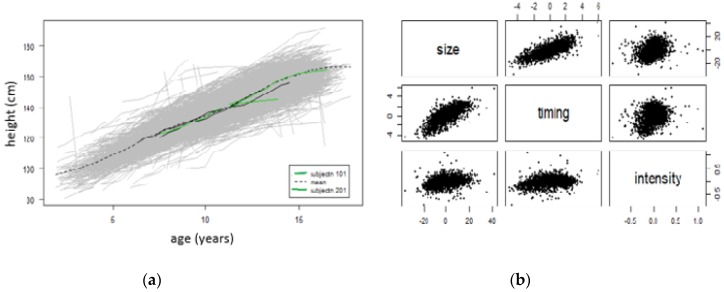
Effects of SITAR adjustment in individual curves for both boys and girls. Effects of SITAR adjestment in individual curves, (**a**) shows the curves in gray as background, plus for the tallest and shortest children before and after adjustment, along with the mean curve (dashed line). For children, subjectn 101 and 201 (**b**) mean height is estimated as 4.4 and 19.3 cm less and greater than average (size) respectively, age at peak height velocity is 1.3 and 4.2 years earlier than average (timing), and mean growth rate is 1.14% and 18% (intensity) respectively. SITAR: Superimposition by Translation and Rotation.

Figure 4 below shows the effects of the SITAR adjustment in individual curves for both boys and girls. For a child (subjectn 101), the mean height is estimated as 4.4 cm less than average (size), age at peak height velocity is 1.3 years earlier than average (timing) and the mean growth rate is 1.14% (intensity). For a child (subjectn 201), the mean height is estimated as 19.3 cm greater than average (size), age at peak height velocity is 4.2 years earlier than average (timing) and the mean growth rate is 18% (intensity).

**Figure 5:**
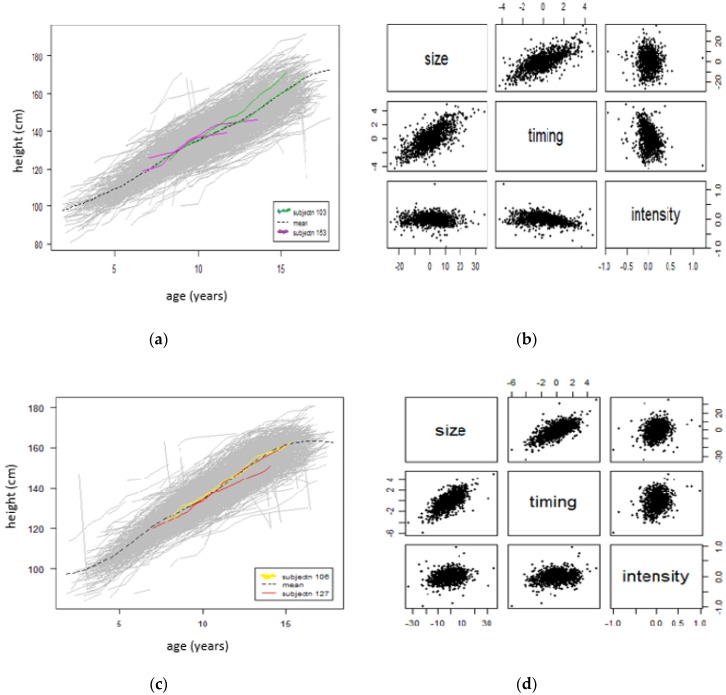
Effects of SITAR adjustment in individual curves for boys and girls (separately). Effects of SITAR adjestment in individual curves, (**a**,**c**) shows the curves in gray as background, plus for the tallest and shortest children before and after adjustment, along with the mean curve (dashed line). For boys, subjectn 103 and 153 (**b**) mean height is estimated as 10.3 and 12.8 cm greater than average (size), age at peak height velocity is 1.7 and 2.8 years earlier than average (timing), and mean growth rate is 19% and 25% (intensity) respectively. For girls, subjectn 106 and 127 (**d**) mean height is estimated as 10.8 and 1.5 cm greater and less than average (size), age at peak height velocity is 2.3 and 0.4 years earlier than average (timing), and mean growth rate is 20% and 0.8% (intensity) respectively. SITAR: Superimposition by Translation and Rotation.

Figure 5 below shows the effects of SITAR adjustment in individual curves for boys (Figure 5a,b) and girls (Figure 5c,d). For a boy (subjectn 103), the mean height is estimated as 10.3 cm greater than average (size), age at peak height velocity is 1.7 years earlier than average (timing) and mean growth rate is 19% (intensity). For another boy (subjectn 153), the mean height is estimated as 12.8 cm greater than average (size), age at peak height velocity is 2.8 years earlier than average (timing) and mean growth rate is 25% (intensity).

For a girl (subjectn 106), the mean height is estimated as 10.8 cm greater than average (size), age at peak height velocity is 2.3 years earlier than average (timing) and mean growth rate is 20% (intensity). For another girl (subjectn 127), the mean height is estimated as 1.5 cm less than average (size), age at peak height velocity is 0.4 years earlier than average (timing) and mean growth rate is 0.8% (intensity).

Table 1 below shows the comparison of the mean curves for both boys and girls combined and boys only and girls separately. It is observed that boys had their age at peak height velocity at 14.45 years compared to the combined 12.51 years (boys and girls), whereas girls had their age at peak height velocity earlier at 11.82 years compared to the combined 12.51 years. In addition, Ellisras rural girls had their age at peak height velocity earlier than Ellisras rural boys did by an estimated 2.63 years.

Comparing the mean peak height velocity, boys only and girls only had their mean peak height velocity later at 6.54 cm/year and 6.52 cm/year, respectively, compared to the combined 6.39 cm/year (boys and girls). In addition, Ellisras rural girls had their mean peak height velocity earlier than Ellisras rural boys did by an estimated 0.02 cm/years.

## 4. Discussion

The Ellisras Longitudinal Study opened the possibilities for understanding the growth variations of children in rural South Africa. The aim of this study was to assess the age at peak height velocity using the SITAR method for both boys and girls. The findings of this study show that all boys and girls have a pubertal growth spurt, a time when they grow considerably faster than before or after. The timing of the spurt varies between the ages 10 and 15 years for both boys and girls.

The mean age at peak height velocity was at 12.51 years and the mean peak height velocity was 6.39 cm/year for both boys and girls (combined). For boys only, the mean age at peak height velocity was 14.45 years and the mean peak height velocity was 6.54 cm/year. For girls only, the mean age at peak height velocity was 11.82 years and the mean peak height velocity was 6.52 cm/year.

The effects of the SITAR adjustment on individual curves for both boys and girls showed that, for a child (subjectn 101), the mean height was estimated as 4.4 cm less than average (size), age at peak height velocity was 1.3 years earlier than average (timing) and the mean growth rate was 1.14% (intensity). For another child (subjectn 201), the mean height was estimated as 19.3 cm greater than average (size), age at peak height velocity was 4.2 years earlier than average (timing) and the mean growth rate was 18% (intensity).

It was observed that boys had their age at peak height velocity at 14.45 years compared to the combined 12.51 years (boys and girls)., whereas girls had their age at peak height velocity earlier at 11.82 years compared to the combined 12.51 years. In addition, Ellisras rural girls had their age at peak height velocity earlier than Ellisras rural boys did by an estimated 2.63 years.

Comparing the mean peak height velocity, boys only and girls only had their mean peak height velocity later at 6.54 cm/year and 6.52 cm/year, respectively, compared to the combined 6.39 cm/year (boys and girls). In addition, Ellisras rural girls had their mean peak height velocity earlier than Ellisras rural boys did by an estimated 0.02 cm/years.

Using the SITAR method, the authors in [14] examined the association between aPHV and hip shape in adolescent boys and girls. They found that mean aPHV was 13.5 years and 11.8 years in boys and girls, respectively. They further concluded that aPHV was strongly related to various components of hip shape, particularly in boys [14].

Cole [7] used the SITAR method to identify the optimal time interval between measurements to summarize individual pubertal height growth. He found that the models for intervals 2–12 months gave effectively identical results for the residual standard deviation (0.8 cm), mean spline curve and random effects (correlations >0.9), showing that there is no benefit in measuring height more often than annually. He concluded that height during puberty needs to be measured only annually and, with slightly lower precision, just four biennial measurements can be sufficient [7].

aPHV and PHV were modeled using the SITAR method and they found that median aPHV was reached approximately three months earlier in youth exposed to maternal diabetes compared with unexposed youth (*p  *< 0.03) [15]. Youth exposed to maternal diabetes had a faster PHV than unexposed youth: exposed girls had a 10.5% greater median PHV compared with unexposed girls, and exposed boys had a 4.0% greater median PHV compared with unexposed boys (*p* < 0.001 for exposure by sex interaction) [15].

O’Keeffe and others tried to better understand if earlier puberty is more likely a result of adiposity gain in childhood than a cause of adiposity gain in adulthood [16]. They found that mean age at peak height velocity was 11.7 years for females and 13.6 years for males. In conclusion, they noted that earlier puberty is more likely a result of adiposity gain in childhood than a cause of adiposity gain in adulthood in females [16]. In males early to puberty, differences in fat mass after puberty were partially driven by tracking of adiposity from early childhood but also greater gains in post-pubertal adiposity [16].

Boys from the UK boys in the Avon Longitudinal Study of Parents and Children had their aPHV at 13.5 years and boys in rural Ellisras had their aPHV at 14.45 years; UK boys were earlier by 0.95 years to have their aPHV than Ellisras boys. UK girls in the Avon Longitudinal Study of Parents and Children had their aPHV at 11.8 years and Ellisras girls had their aPHV at 11.82 years; UK girls and Ellisras girls had their aPHV at the same age of about 11.8 years. Cole [17] used the children from the Edinburgh Longitudinal Growth Study to estimate and compare pubertal growth timing and intensity in height, Tanner stage markers and testis volume. They found that mean APV was 13.0–14.0 years in boys and 12.0–13.1 years in girls, with a between-subject standard deviation of approximately one year [17]. This is comparable to the current study in that Ellisras children do not differ much with Edinburgh children.

## 5. Strength and Limitations

The major strength of the study was the large sample size and the long period of follow up. A limitation of the study was that children were measured twice yearly during the adolescent period as that could mask the growth dynamics as reported in other studies where measurements were taken four times a year [18,19]. However, Boston children were measured twice yearly and yielded similar results to the current study [20]. Furthermore, we did not include the effects of socio-economic status of the children in the analysis.

## 6. Conclusions

In this study, we have analyzed and compared growth variations in height and weight of children in rural South Africa using the SITAR model. Children in rural Ellisras in the Ellisras Longitudinal Study had growth variations that were comparable to other studies such as the Avon Longitudinal Study of Parents and Children and the Edinburgh Longitudinal Growth Study.

## Figures and Tables

**Table 1:** Comparison of mean curves.

	Boys and Girls Combined	Boys Only	Girls Only
Age at peak height velocity (years)	12.51	14.45	11.82
Mean peak height velocity (cm/year)	6.39	6.54	6.52
